# Editorial

**Published:** 1898-06

**Authors:** 


### EDITORIAL DEPARTMENT.

F. E. DANIEL, M. D., Editor.
S. E. HUDSON, M. D., Managing Editor.
A. J. SMITH. M. D., Galveston, Associate Editor.

Published Monthly at Austin, Texas, by Drs. Daniel and Hudson. Subscription price $2.00 a year in advance.

Eastern Representatives: Messrs. Monihan & Fairchild, St. Paul Building, 220 Broadway, New York City.

Official organ of the West Texas Medical Association, the Houston District Medical Association, the Austin District Medical Society, the Brazos Valley Medical Association, the Galveston County Medical Society, and several others.

### EDITORIAL.

PERSONAL.—After an absence of three months, during which time all the work of the JOURNAL fell on our junior—Dr. Hudson—(and we submit he has done it well, especially considering the demands of a large and growing general practice), the "Old Original," ye Senior, takes the helm again; and from the JOURNAL's "conning tower" he will not only guide her, but will have the touching of buttons whereby hot shot, solid shot, bombs and torpedoes will be let loose at the quacks and bogus diploma men, as of yore.

He begs to acknowledge his indebtedness to Dr. A. N. Denton, of Austin, for the able editorials in the May number, which please see.

